# Social Work’s Role in Elder Abuse Research

**DOI:** 10.1093/geroni/igaf122.1480

**Published:** 2025-12-31

**Authors:** Georgia Anetzberger

**Affiliations:** Case Western Reserve University, Cleveland, Ohio, United States

## Abstract

Social work’s interest in understanding and addressing elder abuse predates the 1975 recognition of the mistreatment of older adults as a problem. Indeed, the discipline was the earliest lead in the development and evaluation of adult protective services, today still the dominant intervention for elder abuse. This presentation will explore social work research, both past and present. It will briefly examine collective inquiries to date on adult protective services and other social services directed at elder abuse, with the aim to identify relevant strengths and shortcomings and to offer suggestions for improvement and future research in this domain.

